# A Programmable DNA Biohybrid Material Coupled with Intelligent Cell-Free Computation for Environmental Sensing

**DOI:** 10.1021/acsomega.6c04097

**Published:** 2026-07-21

**Authors:** Hailan He, Ting-Yen Wei, Warren C. Ruder

**Affiliations:** † Department of Bioengineering, 539033 University of Pittsburgh, 300 Technology Drive, Pittsburgh, Pennsylvania 15219, United States; ‡ Department of Mechanical Engineering, Carnegie Mellon University, Pittsburgh, Pennsylvania 15213, United States; § School of Medicine, University of Pittsburgh, Pittsburgh, Pennsylvania 15261, United States; ∥ Institute for Synthetic Biology, University of Pittsburgh, Pittsburgh, Pennsylvania 15260, United States

## Abstract

Programmable materials integrated with intelligent signal processing are essential for developing adaptive and autonomous environmental sensing systems. Here, we present a biohybrid material platform in which environmental DNA is captured by streptavidin (SA)-functionalized microparticles and delivered to a cell-free transcription-translation system for intelligent information processing. The microparticles selectively capture biotinylated DNA from their surroundings, which serve as molecular algorithm inputs for downstream biological computation. DNA immobilized on the particle surface remains transcriptionally active, enabling cell-free systems to produce tunable outputs, including (1) linear responses proportional to DNA input concentration and (2) threshold-dependent switching behaviors achieved through competitive binding. By offloading computation to a cell-free biochemical processor, this approach enables scalable, biocompatible sensing without the need for onboard electronics. This biohybrid design provides a versatile framework for engineering intelligent materials with applications in developing smart tools for healthcare and environmental monitoring.

## Graphical abstract

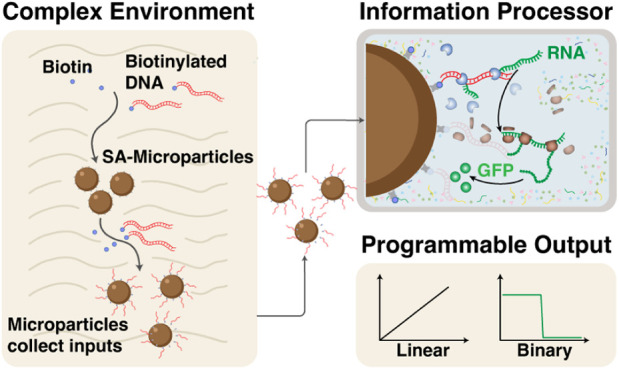

## Introduction

Intelligent materials represent a rapidly evolving class of systems that mimic biological processes by bridging materials science with concepts from chemistry, physics, biology, and engineering.^1,2^ Through the integration of responsiveness, programmability, and multifunctionality, intelligent materials can interact dynamically with external stimuli such as temperature, light, pH, stress, or electromagnetic fields, paving the way for autonomous systems that can learn from and adapt to their surroundings.^1,3–5^ These advances open new possibilities for next-generation applications in medicine, environmental monitoring, and robotics.

Intelligent materials fundamentally rely on two core functional elements: sensors and actuators.^6,7^ Sensors, as the primary interface with the external world, allow materials to perceive their environment.^6^ As the “eyes” of the system, sensors not only recognize environmental cues but also initiate downstream information processing.^8^ Actuators, in turn, respond to the incoming information with actionable outputs, such as signal generation,^9^ conformational transformation,^10^ movement change,^11^ or other adaptive regulations.^12^ Together, these two elements can establish closed-loop adaptive interaction cycles.

Realizing such sensing-actuation behavior at the microscale remains a significant challenge. Traditional approaches often attempt to miniaturize macroscale design paradigms, such as embedding electronic or mechanical logic into smaller devices. For example, Rubenstein et al. designed a swarm of miniature robots that use onboard microcontrollers to share local information and achieve self-assembly.^13^ Ren et al. developed a system that integrated mechanical logic with magnetic composite elastomers to mimic ephyra locomotion.^14^ Park et al. created a biohybrid construct combining a microfabricated gold skeleton with rat cardiomyocytes, enabling light-responsive swimming.^15^ These pioneering studies significantly advanced the field by demonstrating how macroscale designs can be downsized into functional miniature constructs. However, microscale and molecular-scale environments operate under fundamentally different regimes, and strategies effective at larger scales often cannot be directly applied.^3^ Therefore, we must move beyond conventional strategies to new design paradigms tailored for the microscale.

Nature provides abundant inspiration for microscale design principles. Intelligent behaviors of living systems such as taxis, recognition, differentiation, and adaptive memory^16,17^ are governed by molecular programs encoded in nucleic acids.^18,19^ Through transcription and translation, cells exhibit robust capabilities to sense and respond to their surroundings. Advances in synthetic biology now allow these molecular programs to be reengineered, endowing biological systems with functions not observed in nature.^20,21^ Such capabilities open new directions for the design of biohybrid intelligent materials,^22,23^ where sensing, information processing, and actuation are intrinsically embedded at the molecular level, rather than imposed externally.^24,25^

Looking ahead, the development of intelligent materials is expected to increasingly embrace modular design principles, where sensing, processing, and actuation units can be flexibly assembled into multifunctional systems.^3^ Such a strategy not only accelerates prototyping and customization but also enhances adaptability across diverse applications.^26,27^ Within this emerging paradigm, biohybrid approaches offer unique opportunities to realize programmable and evolvable intelligent materials.

In this work, we present a modular, intelligent material system that can sense and respond to environmental cues by integrating DNA, functionalized microparticles, and an off-board cell-free biochemical processor, as shown in Figure 1. The primary motivation is to introduce a new design paradigm that leverages synthetic biology mechanisms to endow materials with advanced computational “intelligence.” Our objective is to validate this theoretical framework and establish the experimental foundation required for future integration into a cohesive intelligent material ecosystem. Previously, we modeled and demonstrated a system that utilized synthetic biology to engineer living cells to program surface chemistry.^28–30^ Using the same competitive binding mechanism, in this paper, sensing microparticles functionalized with streptavidin (SA) selectively captured biotinylated DNA from their environment in the presence or absence of free biotin molecules. Following environmental exposure, these microparticles were collected to relay environmental information to an *E. coli*-derived cell-free gene expression (CFE) system, which served as the central processor. We demonstrated that this processor interpreted the DNA inputs delivered by the microparticles to generate programmable outputs, including linear responses proportional to DNA concentration and threshold-like switching behaviors. This approach highlights how integrating biological computation with modular sensing units can enable new classes of intelligent materials, laying a foundation for future adaptive and autonomous systems.

**Figure 1:**
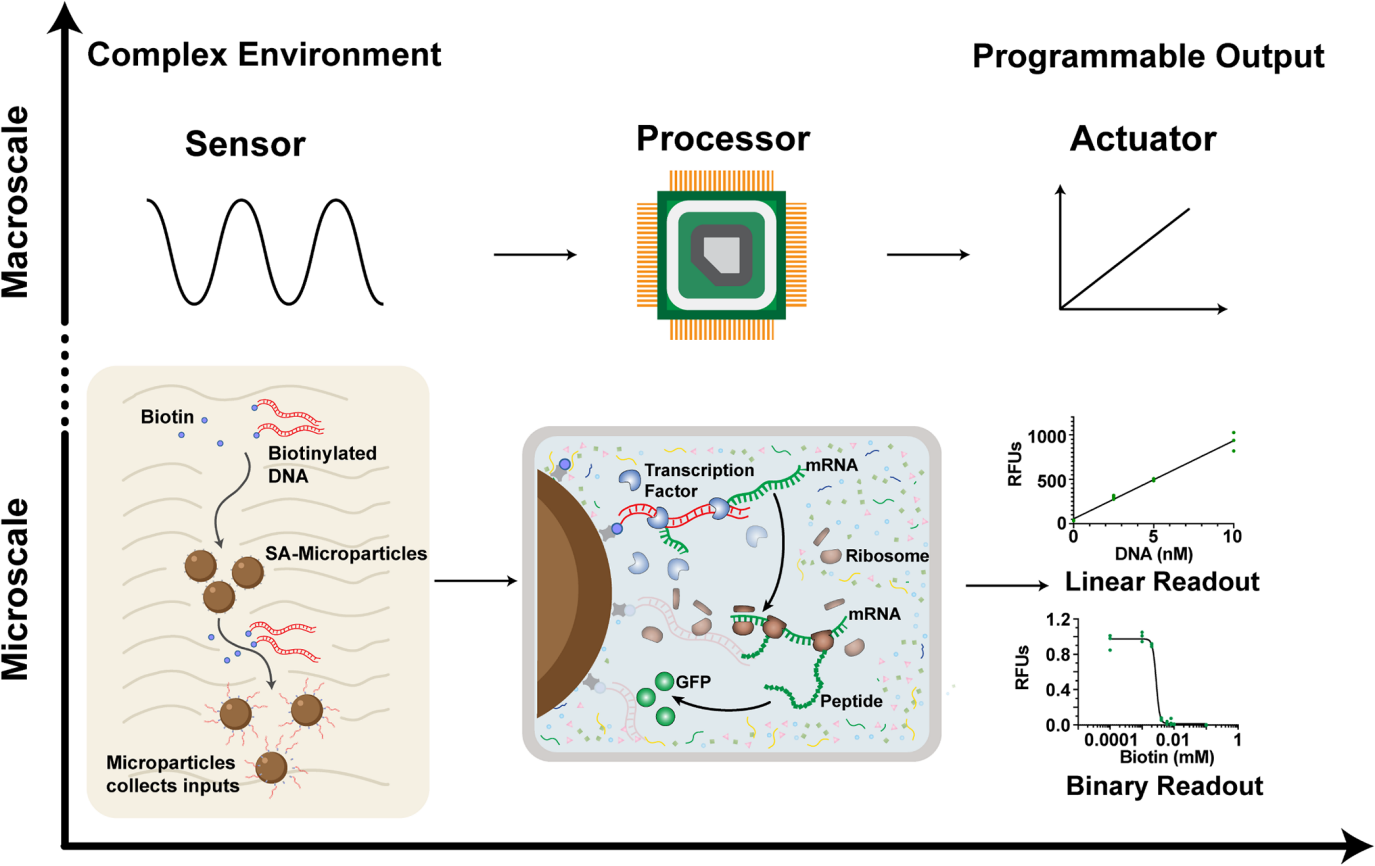
Schematic of a biohybrid material platform where streptavidin (SA) -functionalized microparticles capture environmental biotinylated DNA and deliver it to a cell-free transcription–translation system. The immobilized DNA acts as a molecular input for biological computation, enabling tunable outputs such as linear concentration-dependent responses or threshold-dependent switching through competitive binding. RFU: relative fluorescence units.

## Results

### Material Design and DNA-Based Environmental Sampling

To implement our programmable biosensing system, we employed streptavidin-functionalized microparticles as the host module of the system. The streptavidin on the surface enabled the capture of biotinylated DNA encoding synthetic genetic output modules, allowing the microparticles to collect molecular information from the environment. Because the microparticles interface directly with the environment, we designated them as the “sensors” of the system. DNA captured by these sensors was subsequently processed by a CFE system, which incorporated transcription-translation machinery from crude *Escherichia coli* lysates supplemented with aqueous components.^31^ As the CFE system could use DNA as input and convert the encoded genetic information into a fluorescent output, we referred to it as the “cell-free biochemical processor”. In this context, the “processor” played its role as an input-processing-output module, analogous to information processing systems in engineering.

Before applying this design to environmental detection, we first assessed whether DNA immobilized on microparticle surfaces could still be utilized by the cell-free biochemical processor. The final output from the processor can indicate whether the particles themselves interfered with biochemical processing.

We utilized a linear, biotinylated DNA template encoding an inducible promoter P_LtetO‑1_ and a green fluorescent protein (GFP) reporter gene.^32^ This DNA was immobilized onto streptavidin-coated microparticles and then incubated with the cell-free processor. As shown in Figure 2, GFP expression was observed only in the presence of the DNA, demonstrating that DNA templates were successfully bound to the surface of the microparticles and retained transcriptional accessibility. This result indicated that the microparticles did not hinder cell-free expression activity. These results validate the feasibility of using streptavidin functionalized microparticles as sensors to sense and report environmental nucleic acids.

**Figure 2:**
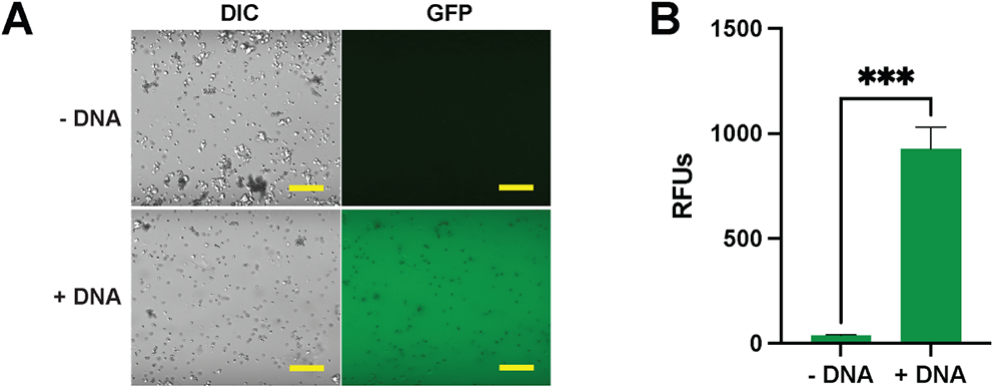
A cell-free system processes DNA immobilized on microparticle surfaces. (A) DIC and fluorescence images showing outputs after DNA processing. Scale bar: 20 μm. (B) Quantification of fluorescence from (A). Data represent the mean ± SD of three replicates. ***P < 0.001. DIC: Differential Interference Contrast. SD: Standard Deviation.

### Quantitative Molecular Sensing by Cell-Free Processor

To explore input-output relationships, we tested whether microparticles could capture environmental DNA at varying concentrations and deliver proportional input to the cell-free processor. Microparticles were exposed to solutions containing increasing concentrations of biotinylated DNA, then separated and incubated in the cell-free processor. As shown in Figure 3A, fluorescence output increased proportionally with input DNA concentration. Linear regression analysis in Figure 3B yielded a high coefficient of determination (R^2^ = 0.9803), confirming the linear relationship.

**Figure 3:**
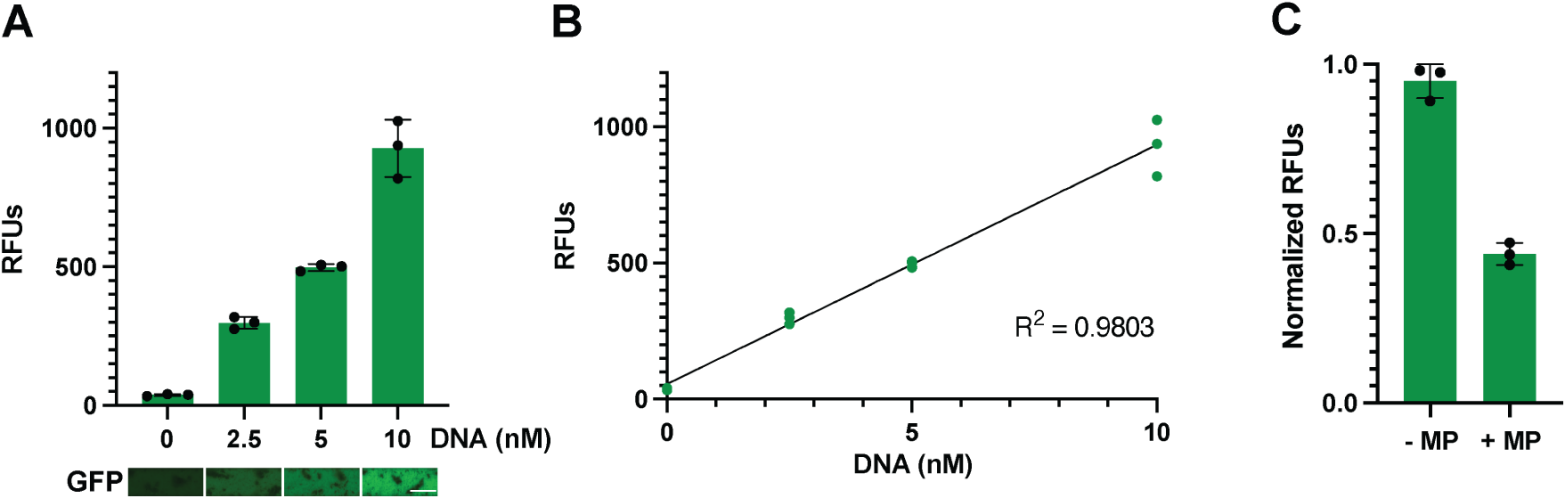
Linear output and binding efficiency. (A) Fluorescence images and quantified outputs from the cell-free system processing DNA input captured by microparticles. Scale bar: 100 μm. (B) Linear regression of DNA input versus output. (C) Normalized translation outputs with and without microparticles (MP). Data represent the mean ± SD of three replicates.

To determine how much DNA was carried away by the sensors from the environment, we assessed DNA capture efficiency by comparing cell-free processor outputs from 10 nM free DNA and microparticle-bound DNA (derived from a 10 nM DNA environment). As shown in Figure 3C, the output for microparticle-bound DNA was approximately half that of free DNA, indicating that around 50% of the input DNA was retained on the microparticles. This result is consistent with prior reports where DNA binding efficiency to streptavidin surfaces ranged between 30–50% for 1–2 kb DNA.^33^ This result demonstrated that our system not only detected the presence of DNA in the environment, but also provided quantitative information about its abundance. This finding confirmed the utility of the cell-free processor for molecular sensing.

These results indicate that the microparticles can reliably sense and deliver proportional molecular information, and that the cell-free system can serve as a biochemical processor for environmental sampling.

While the observed binding efficiency of 50% between biotinylated DNA and microparticles is moderate, this metric does not linearly dictate the ultimate sensitivity or limit of detection (LOD) of the proposed system. The system described here measures the bulk average signal across a volume of microparticles. By relying on macroscopic population-level capture rather than the stochasticity of individual particles, the system effectively integrates the signal over the entire material interface. Furthermore, the deficit in initial target capture could be partially offset by the downstream processing module. The cell-free extract provides inherent enzymatic signal amplification through transcription and translation,^34,35^ ensuring that the overall material ecosystem maintains a robust macroscopic readout even when initial physical capture efficiencies are constrained. Lastly, the efficiency reported here reflects the baseline performance of the current model. In practical applications, the microparticle surface functionalization can be structurally and geometrically optimized with direct nucleic acid probes or target-specific aptamers,^36,37^ allowing us to tune the capture capacity to meet required environmental LODs.

### Binary Output via Competitive Binding Thresholding

Prior work in synthetic biology has demonstrated that systems exhibiting linear analog and binary digital responses to inputs are useful benchmarks for information processing.^38^ Other than the aforementioned linear response capability, we also demonstrate the binary output capability of the cell-free processor.

To implement binary (ON/OFF) decision-making behaviors, we introduced a competitive binding mechanism into the sensing step to modulate the amount of captured DNA. In this assay, streptavidin-coated microparticles were exposed to both biotinylated DNA (the informational input) and excess free biotin (the competitive inhibitor). The competition for streptavidin binding sites altered the amount of DNA captured by the particles, leading to threshold-dependent signal responses in the cell-free processor (Figure 4A–C).

**Figure 4:**
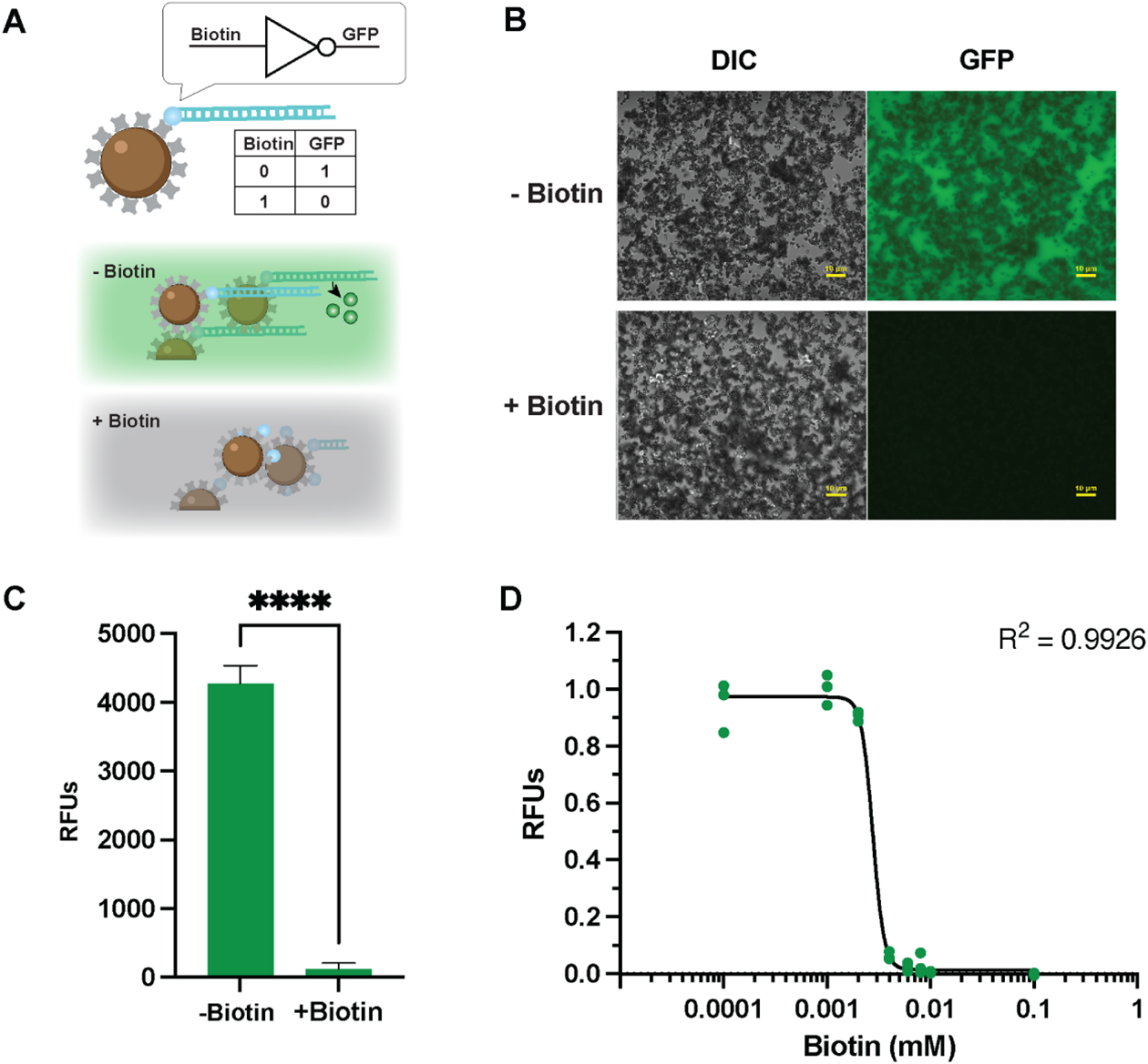
Threshold-dependent binary signal responses in the cell-free system. (A) Schematic of competitive binding between biotin and biotinylated DNA for binary output generation. (B) Representative DIC and fluorescence images showing binary responses at 0.1 mM biotin. Scale bar: 10 μm. (C) Quantification of fluorescence from (B). Data represent the mean ± SD of three replicates. ****P < 0.0001. (D) Dynamic curve of competitive binding illustrating the shifting threshold. The black line is a four-parameter logistic regression fit to the digital-like response.

As shown in Figure 4D, the fluorescence output followed a sigmoidal response, fitting a four-parameter logistic (4PL) regression curve as a function of biotin concentration. The sharp drop in signal was observed at approximately 2.767 × 10^–3^ mM biotin according to the fitting function. This switch-like behavior arises from the kinetics of competitive binding on surfaces.^28,39^ This result demonstrates that the output signal can be modulated by varying the loading density or environmental conditions. Such tunable switching behavior enables digital-like sensing in this system, where defined concentration thresholds of environmental inputs trigger binary outputs. This thresholded response not only demonstrates the programmable nature of our biohybrid material but also provides a functional basis for applications such as noise-resistant environmental monitoring,^40^ threshold-triggered diagnostics,^41^ and on-demand activation of microrobotic systems.^42^

Environmental samples introduce significant noise through both chemical interferents and highly variable target concentrations. To ensure baseline reliability, our system is designed to be highly selective, filtering out nontarget background noise. Our system also employs a dual-layered integration strategy. Physically, deploying a dispersed volume of microparticles integrates the target across the macroscopic reaction space, smoothing out spatial heterogeneity through bulk averaging. Computationally, the downstream cell-free processing module translates this averaged signal through an engineered, digital-like threshold. This nonlinear filter actively suppresses minor concentration fluctuations below the activation point, triggering a definitive binary output only when the bulk concentration strictly exceeds the critical limit.

### Modularity and Universality

Since we employed the well-characterized noncovalent biotin–streptavidin interaction as the sensing module to demonstrate environmental probing, the modular nature of the system suggests that substituting streptavidin-functionalized microparticles of different sizes or surface properties should not impair the cell-free system’s ability to process DNA inputs.

To demonstrate the modularity, we performed DNA sensing experiments using two additional sizes of microparticles. As shown in Figures 5 and S1, microparticles with different diameters all successfully captured environmental DNA and delivered it to the cell-free processor for GFP expression. Notably, the 1.05 μm microparticles, which have hydrophobic surfaces, tended to aggregate in the cell-free extract, producing dense structures under the microscope. In contrast, the 2.8 μm hydrophilic microparticles remained more dispersed.

**Figure 5:**
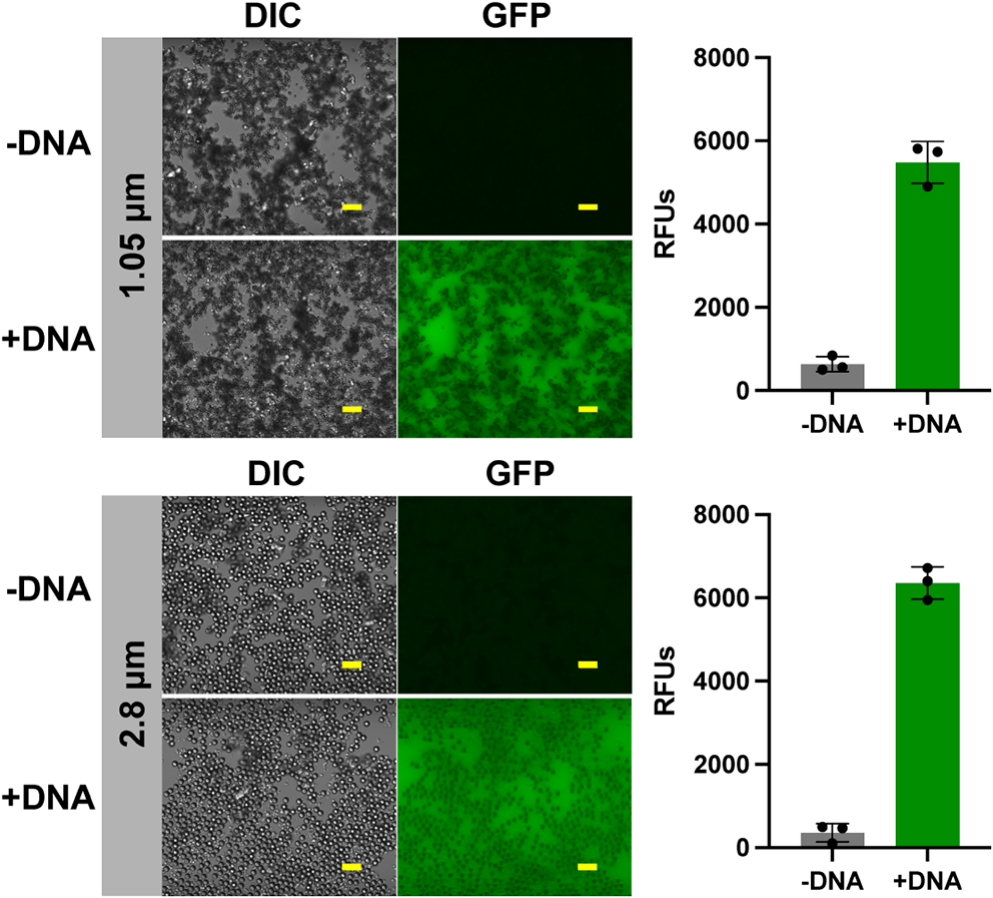
Modularity and universality across microparticle sizes. DIC and fluorescence images on the left with corresponding quantification showing that different-sized particles still generate ON outputs. Scale bar: 10 μm. Data represent the mean ± SD of three replicates.

## Discussion

The proposed biohybrid system presents multiple advantages for practical implementation in future applications. One advantage of the system is that all components operate at the microscale and are amenable to expansion and functional diversification. For example, the strong biotin–streptavidin binding (*K*_d_ = 10^–15^ M) could be harnessed for memory-like functions.^43,44^ Coupled with cell-free extracts, additional functions such as biochemical communication could be introduced, laying the foundation for collective dynamics and swarm behaviors. In addition, our design is modular, where each component, microparticle, binding mechanism, and cell-free processor could be substituted or reconfigured to suit different applications. Lastly, our system can function independently or as part of larger assemblies, where the collective behavior of multiple modules could expand sensing capacity and computational complexity.

In the future, we anticipate that the development of intelligent materials will increasingly embrace modular design principles, in which discrete functional units can be flexibly combined to achieve diverse capabilities. Modular frameworks for our intelligent material, comprising sensing, microparticles, and cell-free processor modules, offer several advantages. First, modular design can accelerate prototyping,^45^ as the modular design allows us to test different sensing mechanisms, employ diverse microparticle materials, and incorporate alternative biochemical processors. Second, modular design enables scalability and adaptability across applications.^46^ For example, in this work, we employed an *E. coli*-based cell-free extract as the processor module. Substituting it with mammalian cell-based extract could broaden the range of detectable DNA sequences and enable more versatile outputs, such as antibody production. Another advantage of modularity is that it is more convenient to customize performance via reconfiguration of modules without redesigning the entire system, thereby reducing cost and complexity.^47,48^ In this work, we used GFP as the output signal, but this output can be substituted with alternative formats such as enzyme production to tailor performance. Additionally, modular principles can help simplify maintenance, since malfunctioning units can be replaced independently.^47^ Finally, modular platforms possess an inherent evolutionary potential, since individual modules can be updated, replaced, or expanded, enabling adaptation to emerging technological demands.

Building on the system’s modular and programmable design, future efforts could focus on developing more advanced intelligent functions. For example, the large surface-to-volume ratio provides abundant sites for functionalization, promising multiplexed molecular recognition, memory and collective computation. Integration of cell-free modules directly within the materials could further advance fully autonomous biohybrid systems. For example, very few wearable sensors could report biomarkers of interest.^49^ Our system offers the potential to detect and report biomarkers in situ by combining the sensing, processing and actuating capability. The microparticles of our system could be adapted to capture various DNA targets from peripheral blood and deliver them to a cell-free chamber. The cell-free processor has the potential to generate an advanced functional response, ranging from the expression of diagnostic reporters (e.g., GFP) to the on-demand synthesis of therapeutic antibodies for interventions in conditions like immune deficiency, which would require carefully designed programs and implantation technology. One study has shown that embedding cell-free extract within hydrogels is feasible for *in vivo* application,^50^ suggesting that it would be possible to adapt our intelligent materials for *in vivo* biosensing. Additionally, modifications to microparticles, such as radiolabeling, could conceivably enable real-time *in vivo* tracing. Ultimately, such intelligent materials that utilize biological intelligence have the potential to perform minimally invasive diagnostic and therapeutic functions in healthcare.

Our platform also has the capacity to adaptively monitor and aid decision-making in complex environmental testing settings. In this study, biotinylated DNA was utilized strictly as a model input to rigorously characterize the signal transduction and processing capabilities of the discrete system components. For future deployment in environmental monitoring, the requirement for target biotinylation could be eliminated by employing direct affinity mechanisms. For nucleic acid targets (e.g., viral or microbial DNA/RNA), the microparticles could be functionalized with complementary single-stranded DNA probes designed for direct sequence-specific hybridization. For non-nucleic acid targets, such as heavy metals or small-molecule contaminants, the material interface could be functionalized with specific aptamers that undergo conformational changes upon target binding to initiate the DNA release and subsequent cell-free computation. Additionally, previous studies have demonstrated the use of DNA-based materials to absorb mercury from aqueous solution^51^ and the application of cell-free systems to detect and report mercury presence.^52^ By integrating both sampling through microparticles and reporting capabilities through cell-free processors, our system could be adapted and expanded to direct detection of mercury in environmental samples and facilitate communication of results to human or computational systems for further analysis and decision-making.

Another promising application of intelligent materials lies in the field of microrobotics, where sensing and actuation remain central research domains.^3,53,54^ Over the past three decades, microscale sensing and actuation have benefited from advances in chemical reactions and physical material changes.^55^ By integrating a cell-free processor, our system introduces a new approach for microscale material design. The biological intelligence of the cell-free processor provides an information processing capability, suggesting the potential for microrobots to function independently.^56^ Moreover, our system could have broader impacts when extended to swarm-level behavior. Prior work has demonstrated precise control of microrobot swarms in terms of their collective shape and trajectory.^57^ By leveraging its modular nature, our system could be expanded to serve as a heterogeneous swarm for diverse applications, either as an independent system or as a modular part of a larger robotic system.

Beyond our specific cell-free architecture, other nucleic acid–based platforms leverage structural programmability for point-of-care diagnostics. For instance, luminescent biosensors engineered on DNA origami scaffolds utilize highly precise nanoscale spatial addressability to detect target nucleic acid sequences.^58^ By converting target recognition into structural conformational changes, these platforms generate luminescent signals robust enough to be quantified directly via smartphone. Furthermore, the broader landscape of intelligent biosensing is rapidly expanding through the integration of molecular recognition with diverse synthetic materials and macroscopic transducers. Recent innovations have successfully coupled biosensing elements with nanozymes, artificial neural networks, flexible paper electrodes, and pressure sensors to achieve multimodal, highly accessible readouts.^16,17,22,23^ While our system embeds information processing intrinsically at the molecular level using cell-free platforms, interfacing such biological processors with these emerging structural scaffolds and flexible electronic transducers represents a logical next step for translating biohybrid materials into user-friendly portable devices.

## Conclusions

Our results demonstrate that streptavidin-functionalized microparticles can act as molecular interfaces, selectively capturing biotinylated DNA from their surroundings and relaying this information to a cell-free biochemical processor. By transferring computation to a cell-free processor, the sensing material circumvents the limitations of onboard electronics, offering an inherently scalable route to information processing. Compared with current intelligent materials that rely on embedded electronics, synthetic polymers, or responsive nanostructures,^59–61^ our biohybrid framework uniquely leverages molecular programmability and the natural computational power of biological machinery. This distinction not only expands the functional space of intelligent materials but also provides a novel route to sensing and decision-making at the microscale, where traditional approaches face significant constraints.

The integration of programmable DNA biohybrid materials with CFE systems possesses several notable features. First, the modular architecture enables straightforward component substitution, allowing individual elements to be replaced or reconfigured as needed. Second, employing an ensemble of microparticles enables the measurement of bulk-averaged signals across the entire reaction volume, providing population-level readouts. These same microparticles are also compatible with flow cytometric analysis, enabling single-particle characterization when higher-resolution data are required. Third, because CFE systems are acellular, they are inherently tolerant of cytotoxic environmental samples that would otherwise compromise cell viability in living-organism-based platforms. Finally, CFE harnesses enzymatic machinery to achieve signal amplification, enhancing detection sensitivity.

Nevertheless, this approach carries certain limitations. CFE systems are compositionally complex and, at present, considerably more costly to produce or procure at scale compared to conventional chemical reagents. Moreover, although enzymatic signal amplification improves sensitivity, the kinetics of transcription and translation introduce an inherent response latency, rendering this platform less suitable for applications that demand instantaneous, continuous real-time monitoring, a capability more readily achieved by electrochemical sensing modalities.

In summary, our system opens new avenues for scalable, multifunctional, and adaptive intelligent materials by bridging molecular programmability, biological intelligence, and modular material design. Building upon this foundational framework, future work will focus on integrating these discrete components and expanding the system’s operational capabilities. By designing novel DNA circuits and employing alternative synthetic biology strategies, we aim to adapt this paradigm for the detection of a broader array of target molecules. Furthermore, we will explore the capacity for higher-order biocomputation within these intelligent materials. Because this architecture relies on a bulk volume of microparticles rather than isolated analytical units, it presents a unique opportunity to engineer complex population-level behaviors. Future iterations will focus on programming more advanced sensing, enabling chemical communication, and facilitating collective computation between particle populations. Ultimately, scaling the complexity of the embedded genetic logic will allow these systems to transition from passive environmental sensors to active, decision-making material ecosystems.

## Experimental Methods

### Biotinylated Linear DNA

Biotinylated linear DNA is a 1161 bp fragment and was generated by performing polymerase chain reaction (PCR) with biotinylated primers from template pTXTL-PLtetO1-deGFP (Arbor Biosciences, Cat No. 502098) and Q5 High-Fidelity PCR Kits (New England Biolabs) on a thermal cycler (Bio-Rad, C1000 Touch Thermal Cycler). The primers were synthesized by Integrated DNA Technologies (IDT) Corporation. See Table S1 for the primers used in this paper. PCR products were purified by using GenCatch Advanced PCR Extraction Kit and quantified by spectroscopy on a microplate reader (BioTek Synergy H1).

### Biohybrid Microparticle Assembly

The molecular algorithms were anchored to microparticle via the SA-biotin interaction. The binding procedure was carried out according to the Dynabeads MyOne Streptavidin T1 manual. Briefly, the microparticles were washed with PBS three times before introducing biotinylated linear DNA and resuspended to 5 μg/μL. Then, every 10^–4^ nmol biotinylated linear DNA was mixed with 10 μL SA-functionalized superparamagnetic microparticles (ThermoFisher, Dynabeads MyOne Streptavidin T1). For competitive binding experiments, every 10^–4^ nmol biotinylated DNA was first mixed with different volumes of biotin solutions and water to the desired biotin concentration in the binding reaction, and then the microparticles were added for SA-biotin binding. After a 15 min incubation at room temperature with gentle rotation, the microparticles anchored with biotinylated linear DNA were separated with magnetic racks and washed with PBS three times. Lastly, the DNA microparticles were resuspended in 2.63 μL nuclease-free water for the following experiments. For Figure 5, equal amounts of particles were used for binding comparison. See Table S2 for information on each particle.

### Cell-Free Reaction

The cell-free platform used in this work was the myTXTL linear DNA Expression kit from Arbor Biosciences. Briefly, stock was removed from a −80 °C freezer and thawed on ice for 30 min. Then, every 2.63 μL of assembled DNA microparticles were added to 7.88 μL of the cell-free extract in a 1.5 mL microcentrifuge tube to ensure enough oxygen was available to the transcription and translation machinery. The tube was then put on a rotator in a 29 °C oven for 16 h at approximately 60 rpm. Although it is recommended that the cell-free extract should not repeatedly be thawed and refrozen, we found one cycle of freeze–thaw does not decrease the transcription and translation expression efficiency significantly.

### Fluorescence Quantification

After the 16-h cell-free reaction, the mixture was put on ice to stop the reaction, and a magnetic rack was used to separate the particles from the solution. Then, 8 μL of the mixture were sampled into a 96-well V-bottom plate for fluorescence quantification. Fluorescence was measured at room temperature in a plate reader (BioTek Synergy H1) using Gen 5 (BioTek, 3.13) with an excitation wavelength of 488 nm, an emission wavelength of 535 nm, and a gain of 50.

### Image Capture and Data Analysis

Brightfield and fluorescent images were captured with a Nikon Ti2A microscope mounted with an Andor Zyla 5.5 sCMOS camera using the NIS-Elements software (Nikon, NIS-Elements AR 5.21.03 64-bit). All experiments were performed in triplicate and reported as mean with standard deviation. Statistical analyses were conducted using GraphPad Prism 10.4.1 for macOS. Detailed methodologies are provided in the Supporting Information.

## Supplementary Material



## Notes

Supporting figures illustrating sensing result with 2.05 μm microparticles, tables with PCR details and microparticles details, along with extended statistical methods, plasmid map and sequences­(PDF)

## Abbreviations

SA: streptavidin
CFE: cell-free gene expression
RFU: relative fluorescence units
GFP: green fluorescent protein
DIC: differential interference contrast
SD: standard deviation
LOD: limit of detection
4PL: four-parameter logistic
PCR: polymerase chain reaction
