# Supplementary material for: A Programmable DNA Biohybrid Material Coupled with Intelligent Cell-Free Computation for Environmental Sensing
Source: ACS Omega. 2026 Jul 21;11(30):45251–9. doi: 10.1021/acsomega.6c04097 (PMC13449214; doi:10.1021/acsomega.6c04097)
Supplement: Supplementary file 1 [file ao6c04097_si_001.pdf]

Supporting Information

A Programmable DNA Biohybrid Material Coupled with Intelligent Cell-Free Computation for Environmental Sensing

Hailan He [a§], Ting-Yen Wei [a§] and Warren C. Ruder [a,b,c,d*]

[a] Department of Bioengineering, University of Pittsburgh, 300 Technology Drive, Pittsburgh, Pennsylvania 15219, United States

[b] Department of Mechanical Engineering, Carnegie Mellon University, Pittsburgh, Pennsylvania 15213, United States

[c] School of Medicine, University of Pittsburgh, Pittsburgh, Pennsylvania 15261, United States

[d] Institute for Synthetic Biology, Pittsburgh, Pennsylvania 15260, United States

* Email: warrenr@pitt.edu

[§] *H.H. and T.-Y.W. contributed equally to this work*

Figure S1. Sensing results of 2.05 μm microparticles.

Figure S2. Plasmid map for pTXTL_PLtetO1-deGFP

Table S1. PCR Information

Table S2. Microparticles Information

Statistical Methods

Plasmid Sequence for pTXTL_PLtetO1-deGFP

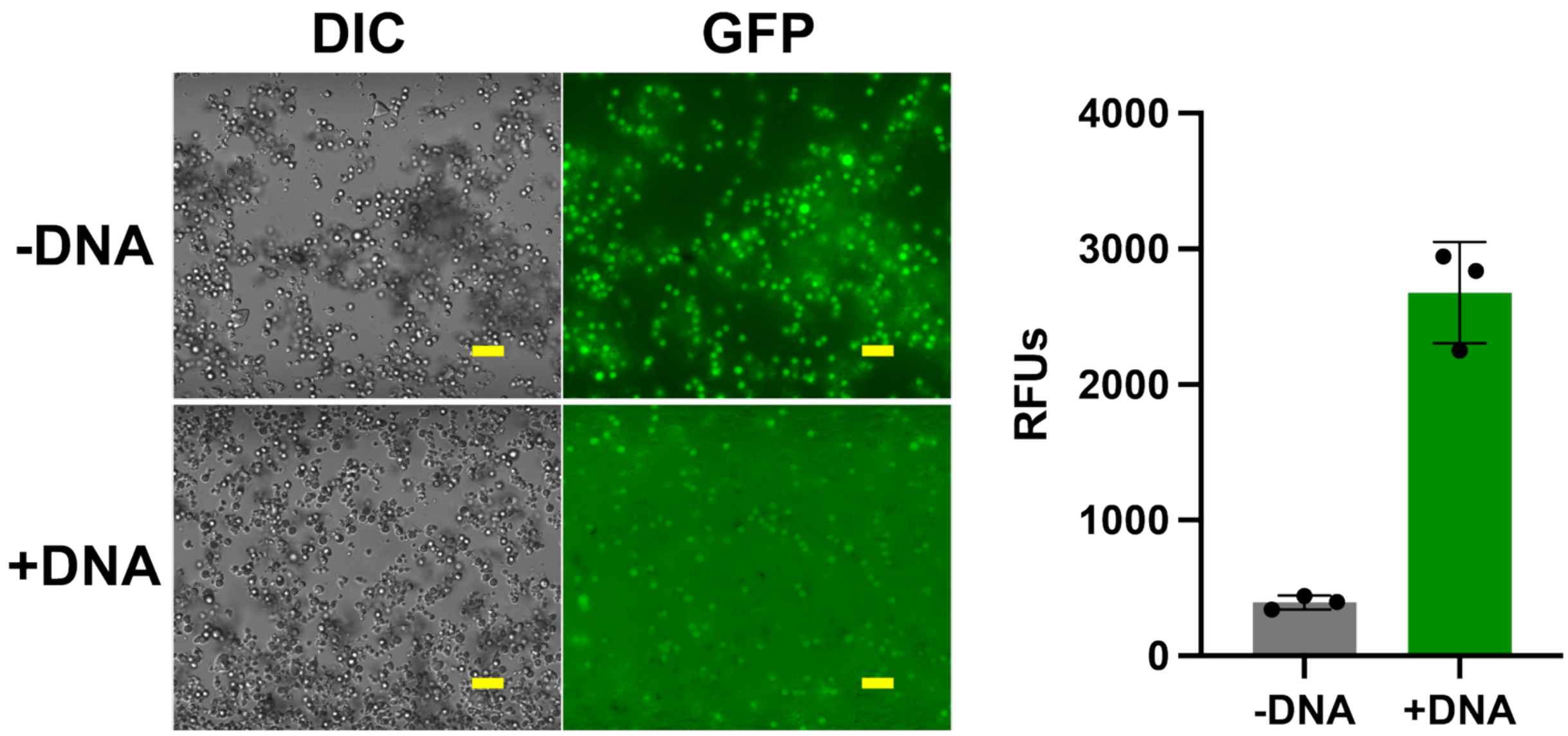

Figure S1. Sensing results of 2.05 μm microparticles. The 2.05 μm microparticles also generated ON outputs upon detecting target DNA in their environment, although they exhibited slightly lower GFP intensity compared to the other two sizes of particles (shown in Figure 5). This reduction of GFP intensity was attributed to the lower biotin-binding capacity of particles from this manufacturer, approximately half that of particles from other manufacturers (see **Table S2**). The observation of green fluorescence from these particles, which primarily exhibit pink fluorescence, was attributed to spectral crosstalk. Scale bar: 10 μm. Data represent the mean ± SD for three replicates.

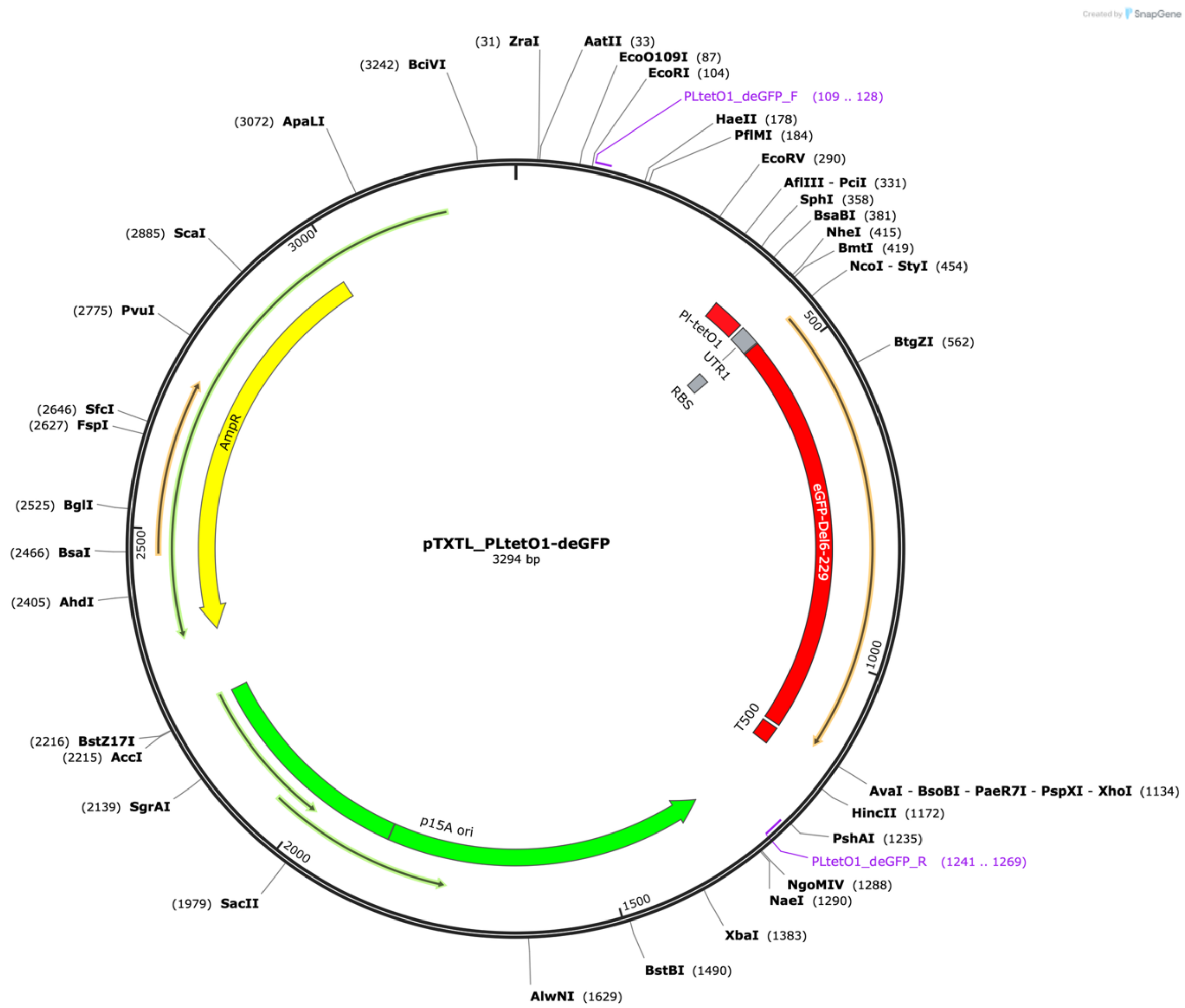

Figure S2. Plasmid map for pTXTL_PLtetO1-deGFP. The plasmid map and the corresponding sequences were generated using SnapGene 8.1.1 software for macOS.

Table S1. PCR Information

Primer Sequences for Linear DNA Template (P_{LtetO1}-deGFP)		
Forward primer		Bio-5'-CTGGCGAATCCTCTGACCAG-3'
Reverse primer		ATGATAAAGAAGACAGTCATAAGTGCGGC
**PCR Reaction Solution**		
Q5 High-Fidelity 2×Master Mix		25 µL
Forward primer (10 µM)		2.5 µL
Reverse primer (10 µM)		2.5 µL
Template P_{LtetO1}-deGFP		1 ng
Nuclease-free water		to 50 µL
**PCR Program**		
Initial denaturation	98 °C	3 min
35 cycles of amplification	98 °C	10 s
	69 °C	30 s
	72 °C	20 s
Final extension	72 °C	2 min
Hold	4 °C	∞

Table S2. Microparticles Information

Size	Name	Catalog #	Binding Capacity for Free Biotin (pmol/mg of beads)
**1.05 µm**	Dynabeads MyOne Streptavidin T1	65601	1100-1700
**2.05 µm**	SPHEROTM Streptavidin Coated Magnetic Fluorescent Particles	FSVM-2058-2	460
**2.8 µm**	Dynabeads M-270 Streptavidin	65305	650-900

Statistical Methods

All following analyses were conducted using GraphPad Prism 10.4.1 for macOS.

For comparison between two groups in Figure 2 and 4, unpaired two-tailed t-tests were performed under the assumption of normally distributed populations with same standard deviation (SD).

The linear regression analysis in Figure 3 was applied to assess the relationship between DNA input and output.

The resulting linear function in Figure 3:

$$Y = 87.85\,x + 55.42$$

For sigmoidal curve fitting in Figure 4, a four-parameter logistic (4PL) model from nonlinear regression was used to find the best function below:

$$Y = Bottom + \frac{Top - Bottom}{\left(\frac{IC_{50}}{x}\right)^{-HillSlope} + 1}$$

The resulting sigmoidal function in Figure 4:

$$Y = 84.74 + \frac{5829 - 84.74}{\left(\frac{0.002767}{x}\right)^{-7.947} + 1}$$

Plasmid Sequence for pTXTL_PLtetO1-deGFP

Note:

1. The highlighted sequence represents the linear DNA template used in this study.
2. Bases shown in red indicate the primer-binding regions.

GCGCACATTTCCCCGAAAAGTGCCACCTGACGTCTAAGAAACCATTATTATCATGAC
ATTAACCTATAAAAATAGGCGTATCACGAGGCCCTTTCGTCTTCAAGAATTCTGGCGA
ATCCTCTGACCAGCCAGAAAACGACCTTTCTGTGGTGAAACCGGATGCTGCAATTC
AGAGCGCCAGCAAGTGGGGGACAGCAGAAGACCTGACCGCCGCAGAGTGGATGT
TTGACATGGTGAAGACTATCGCACCATCAGCCAGAAAACCGAATTTTGCTGGGTGG
GCTAACGATATCCGCCTGATGCGTGAACGTGACGGACGTAACCACCGCGACATGTG
TGTGCTGTTCCGCTGGGCATGCTCCCTATCAGTGATAGAGATTGACATCCCTATCAG
TGATAGAGATACTGAGCACAGCTAGCAATAATTTTGTTTAACTTTAAGAAGGAGATATA
CCATGGAGCTTTTCACTGGCGTTGTTCCCATCCTGGTCGAGCTGGACGGCGACGT

AAACGGCCACAAGTTCAGCGTGTCCGGCGAGGGCGAGGGCGATGCCACCTACGG
CAAGCTGACCCTGAAGTTCATCTGCACCACCGGCAAGCTGCCCGTGCCCTGGCCC
ACCCTCGTGACCACCCTGACCTACGGCGTGCAGTGCTTCAGCCGCTACCCCGACC
ACATGAAGCAGCACGACTTCTTCAAGTCCGCCATGCCCGAAGGCTACGTCCAGGA
GCGCACCATCTTCTTCAAGGACGACGGCAACTACAAGACCCGCGCCGAGGTGAAG
TTCGAGGGCGACACCCTGGTGAACCGCATCGAGCTGAAGGGCATCGACTTCAAGG
AGGACGGCAACATCCTGGGGCACAAGCTGGAGTACAACTACAACAGCCACAACGT
CTATATCATGGCCGACAAGCAGAAGAACGGCATCAAGGTGAACTTCAAGATCCGCC
ACAACATCGAGGACGGCAGCGTGCAGCTCGCCGACCACTACCAGCAGAACACCCC
CATCGGCGACGGCCCCGTGCTGCTGCCCGACAACCACTACCTGAGCACCCAGTCC
GCCCTGAGCAAAGACCCCAACGAGAAGCGCGATCACATGGTCCTGCTGGAGTTCG
TGACCGCCGCCGGGATCTAACTCGAGCAAAGCCCGCCGAAAGGCGGGCTTTTCTG
TGTCAACCGATGCCCTTGAGAGCCTTCAACCCAGTCAGCTCCTTCCGGTGGGCGC
GGGGCATGACTATCGTCGCCGCACTTATGACTGTCTTCTTTATCATGCAACTCGTAG
GACAGGTGCCGGCATGATAAGCTGTCAAACATGAGAATTGCAACTTATATCGTATGG
GGCTGACTTCAGGTGCTACATTTGAAGAGATAAATTGCACTGAAATCTAGAAATATTT
TATCTGATTAATAAGATGATCTTCTTGAGATCGTTTTGGTCTGCGCGTAATCTCTTGC
TCTGAAAACGAAAAAACCGCCTTGCAGGGCGGTTTTTCGAAGGTTCTCTGAGCTAC
CAACTCTTTGAACCGAGGTAACTGGCTTGGAGGAGCGCAGTCACCAAAACTTGTCC
TTTCAGTTTAGCCTTAACCGGCGCATGACTTCAAGACTAACTCCTCTAAATCAATTAC
CAGTGGCTGCTGCCAGTGGTGCTTTTGCATGTCTTTCCGGGTTGGACTCAAGACG
ATAGTTACCGGATAAGGCGCAGCGGTCGGACTGAACGGGGGGTTCGTGCATACAG
TCCAGCTTGGAGCGAACTGCCTACCCGGAACTGAGTGTCAGGCGTGGAATGAGAC
AAACGCGGCCATAACAGCGGAATGACACCGGTAAACCGAAAGGCAGGAACAGGAG
AGCGCACGAGGGAGCCGCCAGGGGGAAACGCCTGGTATCTTTATAGTCCTGTCGG
GTTTCGCCACCACTGATTTGAGCGTCAGATTTCGTGATGCTTGTCAGGGGGGCGGA
GCCTATGGAAAAACGGCTTTGCCGCGGCCCTCTCACTTCCCTGTTAAGTATCTTCCT
GGCATCTTCCAGGAAATCTCCGCCCCGTTCGTAAGCCATTTCCGCTCGCCGCAGTC
GAACGACCGAGCGTAGCGAGTCAGTGAGCGAGGAAGCGGAATATATCCTGTATCAC
ATATTCTGCTGACGCACCGGTGCAGCCTTTTTTCTCCTGCCACATGAAGCACTTCAC
TGACACCCTCATCAGTGCCAACATAGTAAGCCAGTATACACTCCGCTAGGGTCATGA
GATTATCAAAAAGGATCTTCACCTAGATCCTTTTAAATTAAAAATGAAGTTTTAAATCA
ATCTAAAGTATATATGAGTAAACTTGGTCTGACAGTTACCAATGCTTAATCAGTGAGG
CACCTATCTCAGCGATCTGTCTATTTCGTTCATCCATAGTTGCCTGACTCCCCGTCG
TGTAGATAACTACGATACGGGAGGGCTTACCATCTGGCCCCAGTGCTGCAATGATAC
CGCGAGACCCACGCTCACCGGCTCCAGATTTATCAGCAATAAACCAGCCAGCCGG
AAGGGCCGAGCGCAGAAGTGGTCCTGCAACTTTATCCGCCTCCATCCAGTCTATTA
ATTGTTGCCGGGAAGCTAGAGTAAGTAGTTCGCCAGTTAATAGTTTGCGCAACGTTG
TTGCCATTGCTACAGGCATCGTGGTGTCACGCTCGTCGTTTGGTATGGCTTCATTCA
GCTCCGGTTCCCAACGATCAAGGCGAGTTACATGATCCCCCATGTTGTGCAAAAAA

GCGGTTAGCTCCTTCGGTCCTCCGATCGTTGTCAGAAGTAAGTTGGCCGCAGTGTT
ATCACTCATGGTTATGGCAGCACTGCATAATTCTCTTACTGTCATGCCATCCGTAAGA
TGCTTTTCTGTGACTGGTGAGTACTCAACCAAGTCATTCTGAGAATAGTGTATGCGG
CGACCGAGTTGCTCTTGCCCGGCGTCAATACGGGATAATACCGCGCCACATAGCAG
AACTTTAAAAGTGCTCATCATTGGAAAACGTTCTTCGGGGCGAAAACTCTCAAGGAT
CTTACCGCTGTTGAGATCCAGTTCGATGTAACCCACTCGTGCACCCAACTGATCTTC
AGCATCTTTTACTTTCACCAGCGTTTCTGGGTGAGCAAAAACAGGAAGGCAAAATG
CCGCAAAAAAGGGAATAAGGGCGACACGGAAATGTTGAATACTCATACTCTTCCTTT
TTCAATATTATTGAAGCATTTATCAGGGTTATTGTCTCATGAGCGGATACATATTTGAA
TGTATTTAGAAAAATAAACAAATAGGGGTTCC
